# Total Knee Arthroplasty in Patients with Ipsilateral Hip Fusion: Technical Notes and Literature Review

**DOI:** 10.3390/jpm13121705

**Published:** 2023-12-13

**Authors:** Liliana Savin, Tudor Pinteala, Paul Botez, Smaranda Miu, Norin Forna, Dan Mihailescu, Dragos Cristian Popescu, George Enescu, Paul Dan Sirbu

**Affiliations:** 1 Department of Orthopedics and Traumatology, Faculty of Medicine, “Grigore T. Popa” University of Medicine and Pharmacy, 700115 Iasi, Romania; liliana.savin@umfiasi.ro (L.S.); norin.forna@umfiasi.ro (N.F.); dan.mihailescu1@umfiasi.ro (D.M.); dragos.popescu@umfiasi.ro (D.C.P.); enescu_george@d.umfiasi.ro (G.E.); paul.sirbu@umfiasi.ro (P.D.S.); 2 Department Orthopedics and Traumatology, Clinical Rehabilitation Hospital, 700661 Iasi, Romania; paulbotez@yahoo.com; 3 Department of Rehabilitation, Clinical Rehabilitation Hospital, 700661 Iasi, Romania; miu.smaranda@scr.ro; 4 Department Orthopedics and Traumatology, Sf. Spiridon’ County Emergency Hospital, 700661 Iasi, Romania

**Keywords:** total knee arthroplasty (TKA), ipsilateral hip fusion

## Abstract

Numerous studies report the success and outcomes of the total knee arthroplasty (TKA); however, few papers present patients with knee osteoarthritis and ipsilateral hip fusion. One controversy when treating patients requiring a TKA with prior ipsilateral hip fusion is whether to first perform a total hip arthroplasty (THA) of the fused hip, followed by the ipsilateral TKA, or to proceed with the TKA without replacing the hip; studies suggest that the position of the fused hip is a key factor when making this therapeutical decision. In addition, performing a TKA in patients with an ipsilateral fused hip may require modifications to the surgical technique generated by the lack of joint mobility in the hip. We identified 12 studies encompassing 30 patients with hip fusion and ipsilateral TKA in current orthopedic literature, but only six offered insights on patient positioning on the operating table during surgery. This study aims to review the current literature on patients with knee osteoarthritis and prior ipsilateral hip fusion and to present some technical considerations when performing a TKA on a 75-year-old patient with hip ankylosis who underwent a total ipsilateral knee arthroplasty in our clinic.

## 1. Introduction

Hip arthrodesis was considered the surgical intervention of choice in the early 20th century, when treating patients with painful hips caused by osteoarthritis, infection, inflammatory diseases, or traumatic or congenital pathologies [1]. While the initial absence of pain in the fused hip significantly improved patient quality of life, this benefit was not without consequences, as studies have shown that patients with a fused hip started developing pain in neighboring joints such as the ipsilateral knee, spine, or contralateral hip over long periods of time (20 or more years) [1,2,3,4]. Biomechanical studies of the fused hip proved that the lack of movement in the hip joint is compensated by an increase in pelvic transverse and sagittal rotation, increased ipsilateral knee flexion, and an increased mobility of the contralateral hip [2]. In a gait analysis study, Thambyah et al. [5] mentioned that tilting the pelvis induced a pseudo-extension of the hip by increasing lumbar lordosis, whereas the ipsilateral knee presented a decrease in knee flexion moment and a decrease of 95% of the muscle extension moment (similar to a quadriceps avoidance gait). 

The weakness of the quadriceps muscle, commonly seen in this group, can impair their ability to absorb shock while walking. This often leads to more pronounced symptoms associated with knee osteoarthritis and may result in changes in their walking pattern [6,7,8].

Therefore, hip fusion, in time, causes degenerative changes in neighboring joints such as the lumbar spine, sacroiliac joint, ipsilateral knee, and contralateral hip [1,2,4]. 

To date, a small number of papers exist on the management of patients with symptomatic knee osteoarthritis and prior ipsilateral hip fusion, and there is no guideline on how to best manage this situation: to perform a total knee arthroplasty (TKA) or to first convert the fused hip into a total hip arthroplasty (THA) and after to perform the ipsilateral TKA. Studies have mentioned that the position of the fused hip should be considered when deciding between these two therapeutic options. Callaghan et al. [1] clinically measured the hip position in the frontal plane by measuring the angle between the axis of the femur and a line that passes through the anterior superior iliac spine and the femoral vessels (parallel with the midline of the body). Garvin et al. [3] determined the rotational position of the fused hip by measuring the angle between the axis that passes through the second ray and a line perpendicular to the body’s axis in the frontal plane. Romnes et al. [9] performed a TKA in patients with a hip arthrodesis in the “optimal” position (22.5° hip flexion, 5° hip abduction, neutral hip rotation), whereas, in his group of patients considered to have a fused hip in suboptimal position (an average of 38.8° hip flexion, 2.1° hip abduction and 3.3° hip external rotation), the author first converted the fused hip into a THA and subsequently performed the ipsilateral TKA. 

In the rare cases of patients with knee osteoarthritis below a fused hip, surgeons must take into account that the conversion of a fused hip to a THA is a technically challenging procedure with a high rate (up to 54%) of complications such as nerve palsy, early prosthetic failure, heterotopic ossification, and infection [10]. In addition, the rate of hip prosthetic loosening at 10 years is clearly higher in the case of hips with surgical arthrodesis (48.3%) when compared to spontaneous ankylosis of the hip (5%) [11]. Furthermore, given the potential risks of this surgery, most authors suggest that a de-arthrodesis (converting the fused hip into a THA) is controversial if the hip has been fused in an acceptable position (the ideal position for hip fusion is considered 15°–30° of hip flexion (HF), 5°–10° of hip adduction (HA), and 0°–10° hip external rotation (HER) [12]. Therefore, patients with hip fusion (in the correct position), who suffer from end stage osteoarthritis of their ipsilateral knee, can be treated through a TKA only. 

Performing a TKA on patients with a same side hip fusion poses multiple and unique challenges, both in terms of surgical technique and postoperative management as well as possible complications. Current literature offers few data regarding the performance of TKA in patients with ipsilateral hip fusion. This article aims to review the current literature on this subject and present our surgical approach of a patient with end-stage knee osteoarthritis and prior ipsilateral hip fusion.

## 2. Materials and Methods

**Figure 1:**
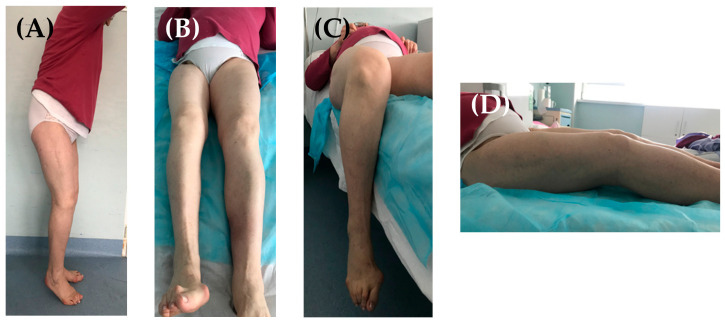
Right hip fusion, with the hip fused in 15° HF (**A**), neutral HA, and neutral HER (**B**). Right knee flexion of 90° (**C**) and 10° of flexion contracture (**D**).

In 2018, a 75-year-old female patient who underwent right hip fusion at the age of 15 due to bacillary osteoarthritis was admitted to our clinic. She complained of severe pain and a significantly decreased range of motion (ROM) in her right (ipsilateral) knee; the patient was ambulating with the aid of crutches. The clinical examination of her right knee (Figure 1) revealed a right knee flexion of 90°, a 15° flexion contracture, and no mediolateral ligamentous instability. Her fused right hip was fixed in a flexion of 15°–20°, neutral abduction/adduction, and neutral internal/external rotation; she did not report any pain/discomfort in this hip. Her leg length discrepancy was measured at 3 cm, and she compensated for it with custom insoles. 

**Figure 2:**
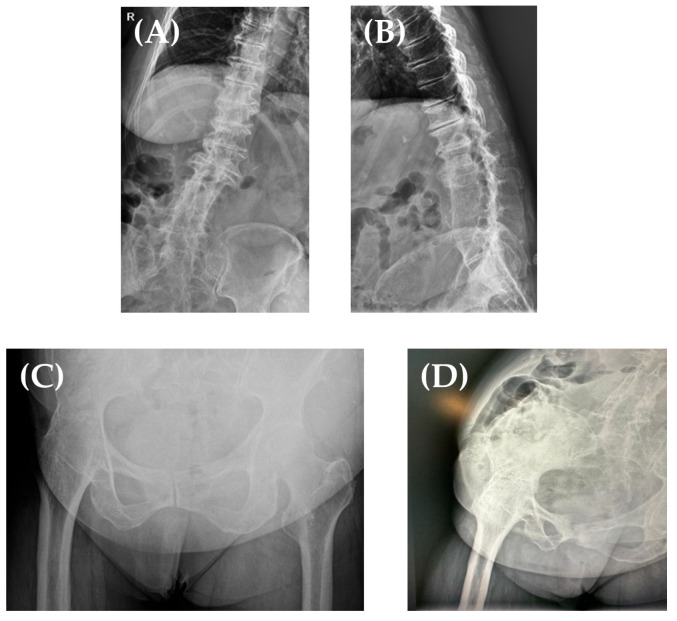
Thoracolumbar kyphoscoliosis, with L2–L4 vertebral block (**A**,**B**). Right hip fused in 15° of hip flexion, neutral hip abduction/adduction, and neutral hip internal/external rotation (AP view—**C**, Lateral view—**D**).

Her X-ray findings (Figure 2) were the following: stage IV Kelgreen and Lawrence right knee osteoarthritis; her right hip was ankylosed in 15° of flexion, neutral abduction/adduction, and internal/external rotation; in addition, she had a thoracolumbar kyphoscoliosis, with L2–L4 vertebral block and severe degenerative changes.

**Figure 3:**
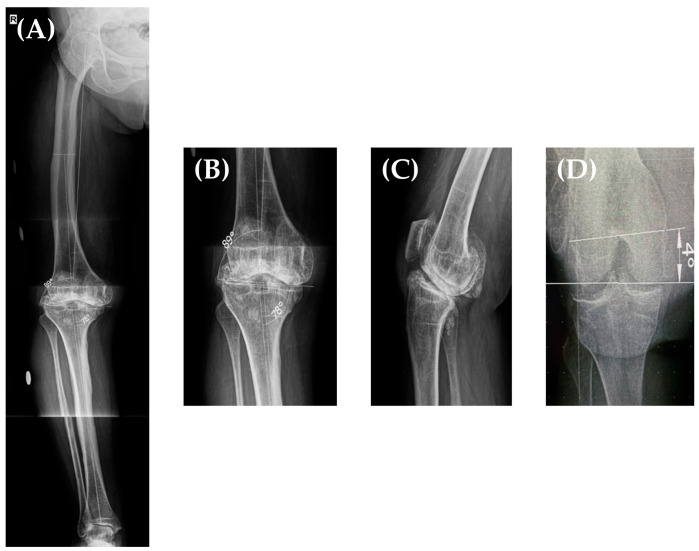
Long leg X-ray of the patient’s right lower limb showing a HKA = 168°, LDFA = 89°, MPTA = 78° (**A**,**B**). Sagittal X-ray (performed at 30° of knee flexion)—increased tibial slope (**C**). Seated view X-ray showing a PCA (femoral torsion) of 4° (**D**).

Taking all of this into account, we decided to perform a total knee arthroplasty on her right knee. Her preoperative planning long leg X-ray (Figure 3A,B) showed a grade II varus deformity of 12° (Hip-Knee-Angle—HKA of 168°), a lateral distal femoral angle (LDFA) of 89°, and a medial proximal tibial angle (MPTA) of 78° [13] with an increased posterior tibial slope and an IM angle (the angle between the femoral anatomical and mechanical axis) of 6°. Furthermore, in our clinic, we routinely perform a “seated view” X-ray which, according to our previous studies [14], helps in determining the distal femoral torsion. Using this special X-ray, where the patient sits on a radiolucent support with the knee flexed at 90°, we determine the posterior condyle angle (PCA) (and thus, the distal femoral rotation) by measuring the angle between the femoral trans-epicondylar axis (a line from the lateral epicondyle connecting it to the sulcus of the medial epicondyle) and the line connecting the posterior femoral condyles (Figure 3D); in this case, the femoral torsion was determined to be 4°.

Before surgery, we obtained written (approved by our hospital’s ethics committee) and verbal consent from our patient to use her clinical history, radiographs, as well as photos from before, during, and after surgery for research/presentation purposes.

In general, when performing a TKA, the patient is supine on the surgical table, and a minimum of 90° of knee flexion is necessary. However, in the case of patients with hip fusion, an ipsilateral knee flexion of 90° is impossible to achieve without making any changes to the surgical table or patient’s position on the operating table. 

## 3. Results

**Figure 4:**
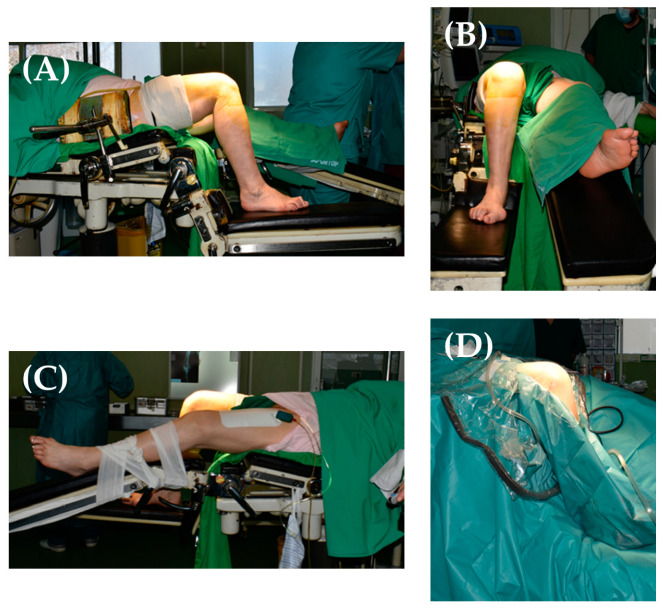
Patient’s position on the operating table and modifications made: thigh support (**A**,**B**); breaking the distal half of or table, which allowed for knee flexion of more than 90° and keeping the distal portion of the table in the normal position (**A**,**B**); positing of the contralateral limb in slight flexion (**C**); operating knee after sterile draping (**D**).

In order to achieve a minimum knee flexion of 90° intraoperatively, we positioned the patient on the operating with the following modifications (compared to a standard supine position when we perform a TKA) (Figure 4): a thigh support fixed in 20° of flexion was used; we broke the ipsilateral distal half of the table, thus causing the knee to be flexed at >90°, and kept the distal arm of the table in the normal position (which provides a solid support the patient’s foot). Therefore, the patient kept her thigh flexion position; when placing the knee at the level of the table break (Figure 4A,B), it could be flexed to more than 90°, and the patient’s foot being placed on the distal part of the surgical table allowed for good structural support (useful during tibial impaction of the prosthetic) (Figure 4A,B). The contralateral limb was positioned in slight flexion (Figure 4C). 

**Figure 5:**
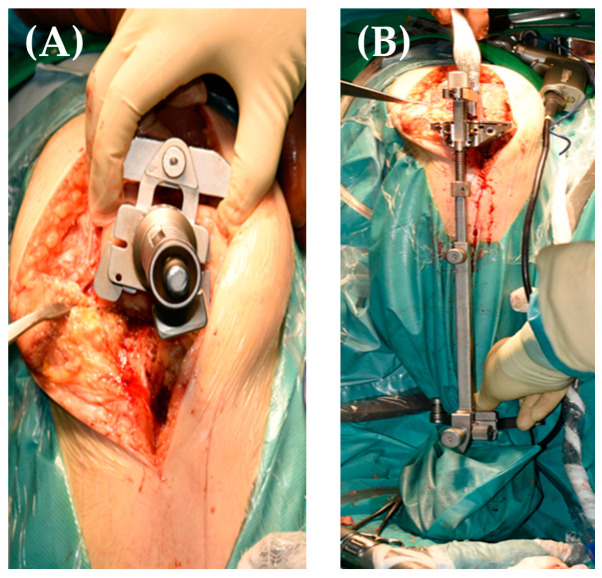
Medial parapatellar approach, placing the intramedullar distal cutting guide with the knee flexed at 90° (**A**), placing the extramedullar tibial cutting guide with the patient’s foot stable on the surgical table (**B**).

A total knee replacement was performed using a cemented postero-stabilized (PS) prosthesis (NexGen) through a medial parapatellar approach, using the extension gap first technique [15]. This type of implant was chosen due to the fact that the patient had competent collateral ligaments, and the ideal flexion-extension gap was intraoperatively obtained using a PS insert. Having the knee flexed and the foot stable on the operating table provided optimal conditions for this surgery. Knee extension was held by a surgical assistant when needed; bone cuts were made according to the preoperative plan (Figure 5A,B). 

**Figure 6:**
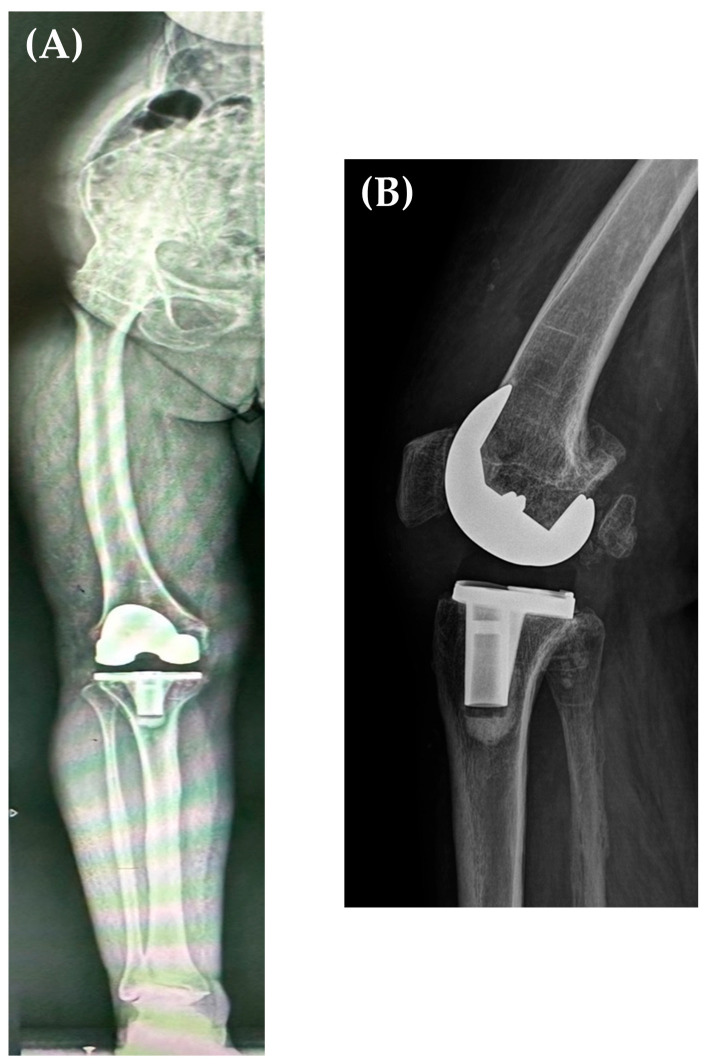
Five years postoperative AP long leg X-ray (**A**) and sagittal X-ray (performed at 30° of knee flexion) (**B**) showing the correct alignment of the femoral and tibial prosthetic components, neutral alignment of the right lower limb, and no component loosening.

Postoperative X-rays revealed adequate positioning and sizing of the prosthetic components and a restored alignment of the lower limb. Personalized rehabilitation was initiated 24 h after the surgery, with knee flexion being recovered at the edge of the bed, and knee extension in lateral decubitus; weight bearing was allowed on the 1st postoperative day, and the patient started walking with the aid of crutches. Subsequent periodic radiological follow-ups at 3, 6, 12 months and annually up to 5 years (Figure 6A,B) did not show any signs of loosening (Figure 3). At her 3-month follow-up, the patient had an active range of motion (ROM) of 110° knee flexion and full extension (in lateral decubitus), walking without any aiding devices, with an increase in KSS from 26 to 84 points and WOMAC increase from 40 to 77.

Searching the PubMed and Google Scholar databases for “total knee arthroplasty” and “ipsilateral hip fusion/arthrodesis/ankylosis”, we found 81 articles, out of which 12 included our criteria: patients with hip fusion/ankylosis/arthrodesis and ipsilateral knee osteoarthritis treated with a total knee replacement. These 12 studies followed and described the total knee arthroplasty in a total of 30 patients with ipsilateral hip fusion (Table 1) [1,3,9,16,17,18,19,20,21,22,23,24]. Callaghan et al. [1] was the first to mention the possibility of performing a TKA in a patient with same side hip fusion, without giving too many details about the respective patients or surgical technique. Out of these 12 articles, only 6 described the changes made to the surgical table during the arthroplasty [18,19,21,22,23,24], and only 2 others mentioned the need for changes [3,17]. Goodman et al. [18] positioned their patients as supine, with the table in Trendelenburg, broke the table at the level of the knee, causing the knee to hang outside; during the surgery, the table was lowered and elevated periodically. Koo et al. [19] placed a sandbag under the ipsilateral buttock, tilted the table towards the operating knee, and hung the knee outside the surgical table. Tang et al. [21] suspended the knee over the distal half of the broken table with the contralateral limb in the lithotomy position. Samborski et al. [22] used multiple mattresses to raise the upper part of the body, the knee being positioned on the surgical table in the Mayo support that offered the possibility of controlled flexion. Ullan et al. [23] positioned the limb in the arthroscopy support. Ashkenazi et al. [24] positioned his patient similarly to Goodman et al. [18]: table in Trendelenburg, broke the distal half of the table, the non-operating knee secured to the table break, and the operated knee bent at the level of the table break. 

In these 12 studies, the patient follow-up period was between 6 and 177 months, and the number of patients included in each study varied from 1 to 9. The postoperative results varied both within the study [3,9] and between studies from fair to excellent, with the most common postoperative complication being the need for multiple closed manipulations due to postoperative knee stiffness [3,9,17].

Garvin et al. [3] followed nine patients, with a mean follow-up period of 7 years (interval between 29 and 177 months), reporting three with excellent results, four as good, one as fair, and one as poor, with seven patients requiring multiple manipulations due to stiffness, and one case finally necessitating an amputation due to a late infection. Rittmeister et al. [16], in a 33-year retrospective study that followed 18 patients with hip ankylosis and ipsilateral knee ostheoarthritis, of which only 3 patients benefited from TKA (follow-up interval between 24 and 86 months) without converting the hip fusion to a THA, reported two poor results and one fair, with 1 patient subsequently requiring knee arthrodesis after three failed revision surgeries. Romness et al. [9] reported three good and one poor outcome in a study that included four patients with TKA and ipsilateral hip fusion, with a follow-up between 2.3 and 10 years. 

Arai et al. [17] placed their patient in a semilateral position; in addition, intraoperatively, they performed a tibial tubercle osteotomy and rectus snip in order to achieve better exposure. The authors present the case of a 50-year-old patient with rheumatoid arthritis who at the age of 36 had a spontaneous right hip fusion (25° HF, 5° HA, 0° HRE) and an ipsilateral arthritic stiff knee (ROM −22° to 30°), where they performed a TKA (Kinemax plus system); intraoperatively, the surgeons decided not to use a PS implant. The authors report immediate postop ROM of the operated knee of 0° to 90°; however, after 1-year post surgery, the patient had a limited ROM of −15° to 30°. Due to this poor result, the authors decided to convert the patient’s ankylosed hip to a THR and unsuccessfully tried a closed manipulation of her knee (due to severe stiffness). Therefore, one month later, they performed a quadricepsplasty and retinacular release on the patient’s right knee; during this surgery, the authors reported to have found severe adhesions and fibrous tissue surrounding the knee prosthesis (to which they attribute the poor outcome of the TKR). One year after the THA, they reported a knee ROM of −15° to 75° and a hip ROM of 0° in extension, 70° in flexion, 30° in abduction, 20° in adduction, and 5° in internal and external rotation [17]. Goodman et al. [18] decided to first perform the patella and tibial cuts followed by the femoral cuts and used NexGen implants. They published two cases: an 87-year-old with their hip fused at 17.5° HF, 5° HA, and 10° HER and a 63-year-old with their hip fused at 15° HF, 5° HA, and 0° HER. They reported good outcomes in both cases at follow-ups (4.7 years and 7.8 years postoperatively) with KSS scores of 83 (33 preop) and 73 (40 preop) in each respective case; however, their second patient had limited flexion due to scarring of their quadriceps. Samborski et al. [22] published the case of a 72-year-old patient, obese and diabetic (type II) with end-stage osteoarthritis of the knee with ipsilateral hip fusion (20° HF, 5 HA°), where they implanted a PS cemented Stryker Triathlon (short-stemmed tibia). During the surgery, the authors encountered no complications; however, after discharge, their patient was diagnosed with chronic kidney injury and required hemodialysis; due to a prolonged immobilization, the patient developed bedsore lesions (heel and dorsal sacrum). At the 2-week postop follow-up, they noted wound healing but noticed a foot drop on the operated side; at the 3-month follow-up, the patient’s wounds were healed, and she was mainly ambulating in a wheelchair with a limited walking distance, aided by a foot drop orthosis and a walker; and, at 6 months after the surgery, the authors reported a passive knee flexion on 0° to 135° [22]. 

Other articles report satisfactory/good surgical outcomes without any complications in their respective follow-ups (Table 1) [18,19,20,21,23,24]. In 2017, de la Hera et al. [20] published two cases; their first case was a 72-year-old patient with a fused hip at 5° HF, 5° HA, and 0° HER, where they performed an ipsilateral TKA (PS NEX-GEN); the authors reported no complications after 11 years postop, with an improvement in KSS from 49 to 65 and increase from 38 to 68 in the WOMAC score. Their second case was a 59-year-old patient with a fused hip at 0° HF, 5° of hip abduction, and 5° HER, who underwent a previous tibia vara osteotomy and later a supracondylar varus osteotomy and subsequently developed end-stage knee arthritis, for which they performed a TKA (LCCK implant with femoral and tibial stems); one year postop, they reported no complications with an increase in KSS from 13 to 88 and WOMAC from 40 to 59. Koo et al. [16] presented the case of a 67-year-old patient with a hip fused at 30° HF and an active flexion of her ipsilateral arthritic knee of 70°. The patient did not suffer any postoperative complications, and the authors established a WOMAC score of 98 at the 6 months postop follow-up with a ROM of the operated knee (PS implant) of 0° to 120°. Tang et al. [21] published the case of a 64-year-old patient with a fused hip at 15° HF, 15° HA, and 20° HER with ipsilateral knee osteoarthritis and previous reduction and internal fixation of her lumbar spine. At the 6 months follow-up, the authors reported no complications, and the patient had a ROM of 0° to 100°. Ullan et al. [23] presented three cases of patients with hip arthrodesis (fused in 15° to 30° HF, 5° to 10° HA, and 0° to 10° HER) and ipsilateral symptomatic knee osteoarthritis, for which they performed a rotational hinge TKA. Their first case was a 72-year-old who had a ROM of the operated knee of −5° to 115°, no sign of complications or loosening, and an increase in the HSS score from 24 (preop) to 72 (postop) at five years postop. The second case was an 81-year-old with hip arthrodesis due to bilateral congenital dislocation who subsequently developed severe valgus knee and complete extension valgus laxity. For this case, the authors chose a constrained rotational hinge implant; at the 3 years follow-up, the authors reported no complications, with a knee ROM of 0° to 100° and an increase in KSS from 45 (preop) to 71 (postop). The third case the authors presented was a 79-year-old patient with hip arthrodesis and a lower limb shortening of 12 cm who developed severe knee osteoarthritis on the same limb (12° genu varum and 11° flexion contracture); in this case, the surgeons decided to implant a hinged TKA (due to the severe malalignment and flexion contracture). One year after surgery, the authors reported no complications, an operated knee ROM of 0° to 110°, and an increase in the KSS score from 32 (preop) to 78 points (postop).

The position of the ankylosed hip was followed in all 12 studies, varying from patient to patient, but generally remaining within acceptable limits and thus permitting a total knee replacement of the ipsilateral osteoarthritic knee. Considering the long period of time in which these studies were published (38 years), the diversified types of prostheses used for these patients is understandable. The patients in the first studies [3,4] benefited from the existing prostheses at the time: guepar, duocondylar, PCA (Porous-coated Anatomic), stabocondylar, or Geomedic; since the 2000s, PS (posterostabilized) [17,18,19,20,22,24], semi-constrained [20], or rotating hinge prostheses [23] have been used. Ashkenazi et al. [24] implanted a PS knee with the use of CAN (computer-assisted navigation); however, the authors acknowledge the limited use of navigation and/or robotic assisted surgery when performing a TKA in a patient with an ipsilateral hip arthrodesis, as the hip center of rotation cannot be determined during surgery due to the lack of hip motion. They solved this issue by registering the femur as a reflected tibia (Table 1).

## 4. Discussion

Nowadays, hip fusion is a rare therapeutical option (primarily due to the advancements in joint replacements) and can be considered in selected cases, such as uncontrolled joint infection or for patients considered too young to be a viable candidate for primary hip replacement [12,25]. The literature suggests that 35–70% of patients complain of back pain, 17–28% develop contralateral hip pain, and 24–57% have ipsilateral knee pain [1,26,27,28] after more than 20 years post hip fusion. In addition, radiographical evidence of ipsilateral knee osteoarthritis is present in over 68% of patients with hip arthrodesis, after several years have passed since surgery [1,9]. 

Treating patients with end-stage knee osteoarthritis and an ipsilateral fused hip provides controversies regarding which joint to replace first. Some suggest that one should first perform a de-arthrodesis of the hip (transform the fused hip into a THA) and then, after a period of time, assess the actual need of a TKA; however, Callaghan et al. [1] reported that 44% of patients have persisting knee pain after a hip de-arthrodesis is performed. Rittmeister et al. [16], in a retrospective study of 18 patients with hip fusion and ipsilateral symptomatic knee osteoarthritis, reported that the most improved HSS knee score (from 33 to 78) were the 4 out of the 18 patients who first underwent a conversion from hip fusion to THA followed by an ipsilateral TKA, whereas the 3 out of 18 patients who “just” underwent a TKA had a mild increase in their HSS knee score (from 35 to 44). Therefore, the authors concluded that hip fusion is a poor prognosis for TKA and that TKA should be performed after hip de-arthrodesis, this therapeutical approach offering the advantage of lower limb mechanical axis realignment and thus decreasing the risk of early implant failure and loosening. Romness and Morrey [9] reported similar HSS knee scores improvements (from 43 to 72 vs. 28 to 72) when they treated 16 patients with same side hip fusion and knee osteoarthritis the following way: 4 patients underwent TKA vs. 12 patients who underwent hip fusion conversion to THA followed by TKA (5.5 years mean follow-up); however, the authors stated that appropriate positioning and good alignment of the hip joints is the most important prognostic factor for TKA. Moreover, Garvin et al. [3] reported similar functional results when comparing nine patients with hip arthrodesis (in a proper position) who underwent an ipsilateral TKA to patients without hip fusion who underwent a TKA. Considering all mentioned above, Cho [29] recommends that when considering treatment options in patients with hip fusion and ipsilateral knee osteoarthritis, the surgeon should consider the position of the hip fusion and the condition of neighboring joints (contralateral hip, spine and sacroiliac joint). Furthermore, Roberts et al. [30] states that the fused hip should be in 25° or less of flexion, 0° abduction, and 5° or less of external rotation in order to obtain good results for the TKA [30]. 

Performing a TKA without first converting the fused hip to a THA poses unique technical challenges due to the lack of mobility in the ipsilateral fused hip, such as patient positioning during surgery, as the usual patient position on the operating table requires certain modifications to obtain adequate knee flexion in order to perform a total knee arthroplasty. Furthermore, surgeons might need some alterations to their surgical technique generated by the lack of joint mobility in the hip for example: Arai et al. performed a tibial tubercle osteotomy and rectus snip for better knee exposure [17]; Goodman et al. started with the patella and tibial cuts followed by the femoral cuts [18]; and Ashkenazi et al., using CAN, made the femoral cut as a reflected tibia without determining the hip center of rotation [24]. 

In general, joint resurfacing requires a profound understanding of normal anatomy and biomechanics [31], and the surgeon should take into account certain surgical technique alterations when performing this kind of surgery in particular patients [32]. 

In our case, the decision to perform a total knee replacement followed the recommendations of available orthopedic literature, our patient presenting a fused right hip in 15° of hip flexion, 0° hip abduction, and 0° hip external rotation. The modification we made to the surgical table allowed us to perform the TKA in safe conditions; furthermore, we feel that maintaining the distal portion of the surgical table in its normal position provides a great benefit, as it stabilizes the foot throughout the surgery, in addition to it being of great help when impacting the tibial component, and thus the surgical technique we used did not require any changes compared to other patients without hip fusion. Moreover, having the operating knee positioned as we did allowed us, during surgery, to flex the knee more than 90°; the knee was kept in extension by a surgical assistant when checking for the extension gap. We believe that these technical modifications aided in the correct implantation of the knee prosthesis, especially the tibial component. Only Samborski et al. [22] maintained a stable foot support in the distal part of the surgical table, having the patient’s body elevated with the help of several mattresses. 

Our patient did not present any ligamentous laxity/instability in the frontal plane, so we decided to use a PS implant without the need to augment bone defects; the patient’s flexion contracture was corrected by performing a posterior capsular release and removal of the posterior osteophytes. Probably, if we were to intervene on this patient now, we would use a cemented short tibial stem in hopes of having a longer implant stability [33,34,35]. The postoperative evolution of our patient was favorable without any complications, and the operated knee mobility at 5 years was preserved (110° of flexion and 0° of extension—measured in the lateral decubitus). 

## 5. Conclusions

To the best of our knowledge, in the current orthopedic literature, few studies report the management of patients with hip fusion and ipsilateral end-stage knee osteoarthritis. Currently, there are no clear guidelines for how to best manage this condition. In our opinion, patients with this particular pathology can benefit from a total knee arthroplasty with good to excellent outcomes, if the hip is fused in an optimal position. We consider that preoperative clinical and radiological planning is crucial in determining the therapeutic approach (performing a TKA and keeping the hip fused vs. converting the fused hip into a THA followed by TKA) and selecting the appropriate knee implant (taking into consideration collateral ligaments’ stability and bone defect). In addition, the surgeon should consider patient positioning modifications in order to obtain more than 90° of knee flexion and good stability of the joint during surgery (despite the lack of hip mobility). Even though our study focuses on a single case, we believe our surgical table modification can assist other surgeons dealing with advanced knee osteoarthritis and ipsilateral hip fusion.

## Figures and Tables

**Table 1:** Findings from the reviewed literature of patients with TKA and ipsilateral hip fusion.

No. Crt.	Author	No. of Pts	Hip Fusion Position	HSS Knee Score	Results	Follow-Up Interval	Type of Prosthesis	Complications	Patient Position during Surgery
1	Callaghan (1985) [1]	2	-						-
2	Garvin (1989) [3]	9	37° HF 1° HA 8° HER	-	3 excellent 4 good 1 fair 1 poor (infect)	29–177 months	1 duocondylar 1 guepar 3 total condylar 4 PS	7 multiple manipulations 1 Guepar prosthesis—quadriceps rupture, late infection (7 years), amputation	Bolster to elevate the hip fused
3	Romness (1991) [6]	4	20°–30° HF 0°–10° Abd 0° HER	43.5 to 72.1	3 good 1 poor	2.3–10 years	7 Kinematic 3 PCA 1 Kinematic stabilized 1 Stabocondylar 1 Tavernetti 1 Geomedic	1 manipulation	-
4	Rittmeister (1999) [13]	3	20°–30° HF 10° HA 10°–20° HER	36 to 44	2 poor 1 fair	24–86 months	-	1 deep infection, three revision, and arthrodesis	-
5	Katsumitsu Arai (2001) [14]	1	25° HF 5° HA 0° HER		Limited ROM	1 year	PS	Fused hip converted to THA, Knee manipulation Quadriceps plasty	Semilateral position
6	Goodman (2014) [15]	2	15°–17.5° HF 5° HA 0°–10° HER	33 to 83 40 to 78	satisfactory	4.7–7.8 years	PS NexGen	-	Table in Trendelenburg Knee at the level of the break in the table Flexed the table foot to 90°
7	Koo (2015) [16]	1	30° of hip flexion Neutral hip adduction	WOMAC score 98	Excellent	6 months	PS	-	Sandbag placed under the ipsilateral buttock Table tilted about 20° towards the operated knee Hang the leg over the table
8	De la Hera (2017) [17]	2	0°–5° HF 5° HA–5° Abd 0°–5° HER	49 to 65 13 to 88		1–11 years	1 PS NexGen 1 semi-constrained LCCK	-	-
9	Tang (2019) [18]	1	15° HF 15° HA 20° HER	-	Good	6 months	-	-	Knee suspended over the broken table Contralateral leg in lithotomy position
10	Samborski (2020) [19]	1	20° HF 5° HA 20° HER	-	-	6 months	PS Triathlon with short tibial stem	Respiratory failure, acute kidney injury, hemodialysis, decubitus ulcers, foot drop	Multiple operating table mattress pads under the upper body Remove the pads under the operated leg De Mayo Surgical leg positioner
11	Ullan (2021) [20]	3	15°–30° HF 5°–10° HA 0°–10° HER	24 to 72 45 to 71 32 to 78	Satisfactory	1–5 years	Rotating hinge	-	Knee suspended with arthroscopy leg holder
12	Ashkenazi (2023) [21]	1	5°–10° HF 10° HER	-	-	2 weeks	Robotic assisted surgery PS	-	Table in Trendelenburg, broke the distal half of the table, and secured the contralateral leg to it. The operated knee was placed at the level of the table break (similar to Goodman et al.)

Pts = Patients; HF = Hip Flexion; HA = Hip Adduction; HER = Hip External Rotation; Abd = Abduction; PS = Postero-Stabilized; ROM = range of motion; manipulation = closed manipulation under anesthesia.

## Data Availability

The dataset used and/or analyzed during the current study is available from the corresponding author on reasonable request.
